# Classification and incidence of cancers in adolescents and young adults in England 1979–1997

**DOI:** 10.1038/sj.bjc.6600647

**Published:** 2002-11-12

**Authors:** J M Birch, R D Alston, A M Kelsey, M J Quinn, P Babb, R J Q McNally

**Affiliations:** Cancer Research UK, Paediatric & Familial Cancer Research Group, Royal Manchester Children's Hospital, Stancliffe, Hospital Road, Manchester M27 4HA, UK; Central Manchester and Manchester Children's University Hospitals, NHS Trust, Pathology Department, Royal Manchester Children's Hospital, Hospital Road, Manchester M27 4HA, UK; National Cancer Intelligence Centre, Office for National Statistics, 1 Drummond Gate, London SW1V 2QQ, UK

**Keywords:** adolescent cancers, young adult cancers, cancer incidence, cancer trends, classification of cancers, cancer registries

## Abstract

Cancer patients aged 15–24 years have distinct special needs. High quality cancer statistics are required for service planning. Data presented by primary site are inappropriate for this age group. We have developed a morphology-based classification and applied it to national cancer registration data for England 1979–1997. The study included 25 000 cancers and 134 million person–years at risk. Rates for each diagnostic group by age, sex and time period (1979–83, 1984–87, 1988–92, 1993–1997) were calculated. Overall rates in 15–19 and 20–24-year-olds were 144 and 226 per million person–years respectively. Lymphomas showed the highest rates in both age groups. Rates for leukaemias and bone tumours were lower in 20–24 year olds. Higher rates for carcinomas, central nervous system tumours, germ-cell tumours, soft tissue sarcomas and melanoma were seen in the older group. Poisson regression showed incidence increased over the study period by an average of 1.5% per annum (*P*<0.0001). Significant increases were seen in non-Hodgkins lymphoma (2.3%), astrocytoma (2.3%), germ-cell tumours (2.3%), melanoma (5.1%) and carcinoma of the thyroid (3.5%) and ovary (3.0%). Cancers common in the elderly are uncommon in adolescents and young adults. The incidence of certain cancers in the latter is increasing. Future studies should be directed towards aetiology.

*British Journal of Cancer* (2002) **87**, 1267–1274. doi:10.1038/sj.bjc.6600647 www.bjcancer.com

© 2002 Cancer Research UK

## 

The National Health Service Cancer Plan, aims to improve survival rates by delivering the best and most appropriate treatment for each individual patient (Department of Health, 2000). An important element in achieving this aim is the availability of high quality population-based cancer statistics. In the United Kingdom such data are available through national cancer registration (Quinn *et al*, 2001; Walsh *et al*, 2001; http//www.show.scot.nhs.uk/isd/cancer/cancer.htm). One important group of cancer patients with special needs is those diagnosed during adolescence and young adulthood. While a co-ordinated approach to the treatment of cancers in children has been established for many years adolescents and young adults have fared less well (Bleyer, 2002). Cancers in this age group, even if cured, can have a devastating effect on future life including the development of second cancers (Bleyer, 2002). In order to develop services tailored to their needs, it is necessary to define the extent and nature of the patient population through precise analyses of relevant population-based data.

Cancer incidence data are usually presented in terms of primary site according to the International Classification of Diseases (ICD) (World Health Organisation, 1975, 1992; Parkin *et al*, 1997). This is broadly satisfactory for late age of onset, cancers which are mainly carcinomas, but in young people carcinomas are much less important numerically. For epidemiological and service planning purposes, data on cancers in young people should be presented mainly in terms of morphology. The aims of the present study were to develop a diagnostic classification scheme appropriate to cancers in young persons and to apply the scheme to national cancer registration data to give an accurate and balanced picture of cancers in this age group.

## METHODS

Anonymised individual level national cancer registration data for the years 1971 to 1992 were obtained on CD-ROM (Quinn *et al*, 1999). More recent data up to 1997 were supplied directly by the Office for National Statistics (ONS). It has been demonstrated that there was probably some under-registration during the early 1970s but, from the late 1970s onwards registration has been largely complete and consistent (Quinn *et al*, 2001). Because of these considerations, data for the years 1979 to 1997 were analysed for the present study. Furthermore, in earlier years, data were coded in MOTNAC (American Cancer Society, 1968) which translates very imperfectly into the proposed classification. From 1979 to 1994 data are coded by ICD-O first edition (World Health Organisation, 1976) and ICD ninth revision (World Health Organisation, 1975) and from 1995 onwards by ICD-O second edition (Percy *et al*, 1990) and ICD tenth revision (World Health Organisation, 1992). National population estimates by single year of age, sex and calendar year were supplied by the Population Estimates Unit, ONS.

### Construction of the classification scheme

For registered cancers in the age range 15 to 24 years, frequency tables by individual ICD-O M-code were produced. These tables were used to formulate groupings of M-codes to create main diagnostic groups. In defining the groups, the following principles were applied: (1) the groupings should reflect the numerical importance of specific cancers; (2) common cancers should be specified as individual categories; (3) rare tumours of particular interest should also form specific categories; (4) categories should be based principally on morphology with biologically similar tumours classified together; and (5) diagnostic categories should be hierarchical to allow maximum flexibility in analyses.

### Statistical analyses

Eligible cases included all malignant tumours, except carcinomas of skin, occurring in England from 1979 to 1997 in individuals aged 15 to 24 years. *In situ* cancers and neoplasms of uncertain behaviour were excluded. Scrutiny of the data for non-malignant central nervous system (CNS) tumours suggested that there were variations in registration practices between regional cancer registries, so only malignant CNS tumours were included in the analyses.

Individual cancer registrations were classified by; cancer type, age group (15 to 19, 20–24 years), sex and time period (1979–83, 1984–87, 1988–92, 1993–97). These time periods were chosen to give the most even distribution of person–years at risk. The person–years at risk for each sub-group were calculated from the population data. Age group, sex and diagnostic period specific annual incidence rates per million population were calculated. Age-specific rates for the 15–19-year-olds and 20–24-year-olds were calculated. An age-standardised rate was calculated at each time period, using direct standardisation to the world population (Parkin *et al*, 1997). A combined rate for the entire time span was obtained using a weighted average of the separate standardised rates (Blair and Birch, 1994). The significance of variability by sex, age group and time period was assessed using Poisson regression. Changes in the incidence rate over time were assessed after taking into account variability in cancer rates by age and sex. It was assumed that the changes in incidence rates over time were consistent. All calculations were performed using the statistical package GLIM 4 (Francis *et al*, 1993).

## RESULTS

### The classification scheme

Ten main cancer groups were defined, each of which (except group 10, unspecified) is divided into sub-groups giving 32 in all. Fifteen of the sub-groups are further sub-divided, thereby providing three levels of classification. The classification scheme is shown in Table 1

**Table 1:**
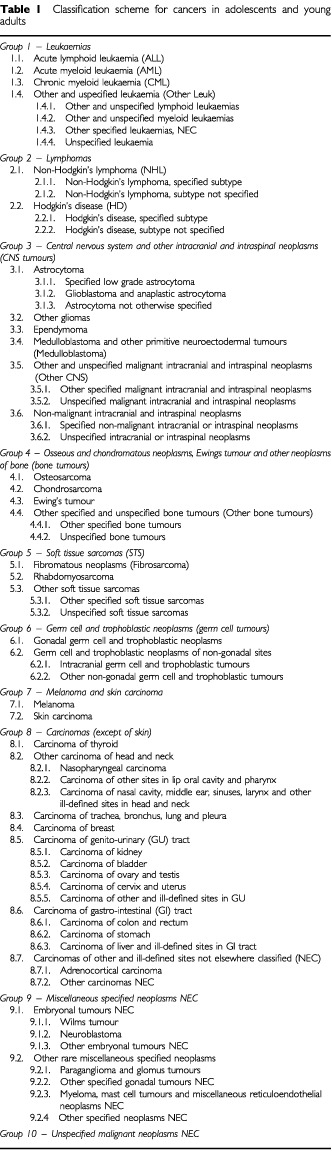
Classification scheme for cancers in adolescents and young adults

. Each main group and each level of sub-group are specified by combinations of ICD-O (eds.1 & 2) M-codes and topography codes (T code). For example, cases with the morphology code 8800/3 (sarcoma not otherwise specified) are classified with bone tumours if combined with a T-code in the range C40.0–C41.9 (bones, joints and articular cartilage) but if combined with any other T-code, these cases are classified with soft tissue sarcomas. The group-specific M and T code allocations together with respective algorithms for selecting tumour groups are given at: (http://www.biomed2.man.ac.uk/crcpfcrg/CRUKPFCRG/PFCRG.htm).

As in published childhood cancer classification schemes based on ICD-O first and second editions (Birch and Marsden, 1987; Kramárová and Stiller, 1996), leukaemias, lymphomas and CNS tumours constitute the first three major groups. However, non-CNS embryonal tumours including neuroblastoma, retinoblastoma, Wilms' tumour and hepatoblastoma, which form the major components of groups IV, V, VI and VII in the childhood classifications, are extremely rare over the age of 15 years. All of these tumours are therefore grouped together under Miscellaneous Specified Neoplasms NEC (Table 1, Group 9).

Bone and soft tissue sarcomas, as in childhood, represent numerically important malignancies in adolescents and young adults and these tumours form Groups 4 and 5. In the childhood cancer classifications, extra-skeletal Ewing's tumour and other extra-skeletal peripheral primitive neuroectodermal tumours are classified with soft tissue sarcomas but in the proposed classification for cancers in adolescents and young adults, we have grouped all Ewing's and related tumours together regardless of coded primary site. There are two reasons for this: firstly, morphologically and biologically such tumours are all similar and are characterised by the presence of the Fli-l;EWS fusion gene (Parham, 1996) and secondly, even with full clinical information it is often difficult to determine the origin of such tumours and not possible to ascertain whether a tumour is arising in bone and invading soft tissue or vice versa. In practice the extra-skeletal tumours of the Ewing's family are rare and make little difference to the overall rates.

A similar argument can be made for grouping all malignant fibrous histiocytomas (MFH) together. Very rarely, MFH can arise in bone but in the proposed classification all cases classified morphologically as MFH are grouped with fibromatous neoplasms (Group 5.1). Malignant peripheral nerve sheath tumours (MPNST) are included with other specified soft tissue sarcomas (Group 5.3.1). In the childhood classifications, MPNST was included with fibromatous neoplasms but this is inconsistent with current classifications and with the histogenetic origin of these tumours (Weiss and Goldblum, 2001).

Group 6 comprises malignant germ cell tumours. Although germ cell tumours form a separate group in the childhood classifications they represent approximately 3% only of total childhood cancers (Mcnally *et al*, 2001a). In the 15–24 year age group, germ cell tumours are much more numerous and in the present series, constitute approximately 14% of the total cancers. Similarly, malignant melanoma, which is very rare in children, represents a important group in adolescents and young adults both numerically and clinically and is included in Group 7. One of the main failings of the childhood cancer classification with respect to its application to young adult cancers is the lack of detail and the inappropriate sub-groupings of carcinomas. The proposed classification employs a more detailed treatment of carcinomas (Group 8).

Table 2

**Table 2:**
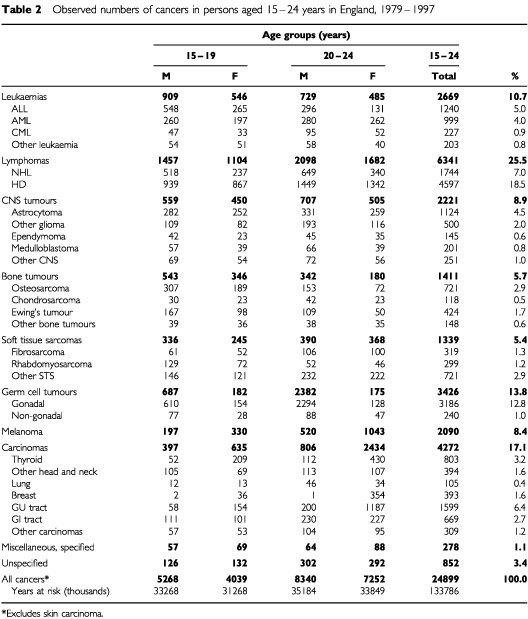
Observed numbers of cancers in persons aged 15–24 years in England, 1979–1997

shows the numbers of cases included in the study classified according to main group and first level of sub-group, by age group and sex. There were almost 25 000 eligible cases and the study covered nearly 134 million person–years at risk.

### Cancer rates by age and sex

Table 3

**Table 3:**
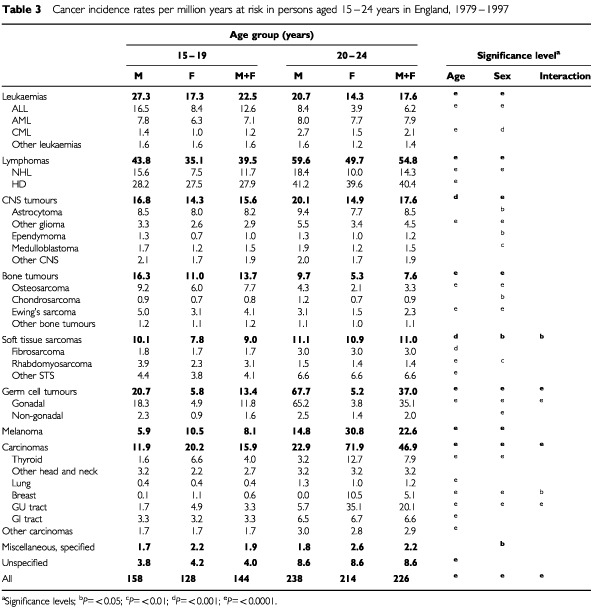
Cancer incidence rates per million years at risk in persons aged 15–24 years in England, 1979–1997

shows the incidence rates per million person–years for each age group in males and females by diagnostic group. Overall rates in both males and females were significantly higher at 20 to 24 years than at 15 to 19 years (*P*<0.0001) and rates were significantly higher in males than in females in both age groups (*P*<0.0001). There was however, some variation in the pattern according to diagnostic group. Among leukaemias, rates were higher in males than in females. The incidence of ALL in 15 to 19 year olds was twice that in 20 to 24 year olds. ALL is one of only two groups to show this pattern (see below). For AML the rates are similar in both age groups, but CML has higher rates at older ages. Rates for lymphomas were significantly higher in the older age group particularly for HD. For NHL, rates in males were significantly higher than in females but rates for HD were similar in both sexes.

Rates for CNS tumours were higher for males aged 20 to 24 years than 15 to 19 years but not for females. The only individual sub-group to show significantly higher rates in the older age group was the ‘other gliomas’. CNS tumours had higher rates in males than in females. Bone tumours form the other group showing a fall in rates at older compared with younger ages. This pattern was apparent in both osteosarcoma and Ewing's tumour. Rates for bone tumours were significantly higher in males than in females. Overall, soft tissue sarcomas had higher rates among the 20 to 24 year olds, but rates for rhabdomyosarcoma were significantly higher at younger ages. The biggest difference in rates between the younger and older age groups was seen for germ cell tumours in males (20.7 and 67.7 respectively). This was entirely accounted for by testicular tumours.

In contrast to the first six cancer groups, where rates were higher in males, rates in the last four groups were consistently higher in females. Highly significant increases in rates between the younger and older groups were seen for melanoma and carcinomas. This pattern was present for all the sub-groups of carcinomas but was most marked for carcinomas of breast and GU sites, where the ratio between rates in the older and the younger groups were 8.7 and 6.1 respectively. GU carcinomas were further examined by sub-type: differences in rates between age groups were 3–4-fold for carcinomas of kidney, bladder and ovary but the most marked difference was in carcinoma of the uterine cervix which increased from 0.8 per million in 15 to 19 year olds to 21 per million in 20 to 24-year-olds.

### Temporal trends in incidence

Table 4

**Table 4:**
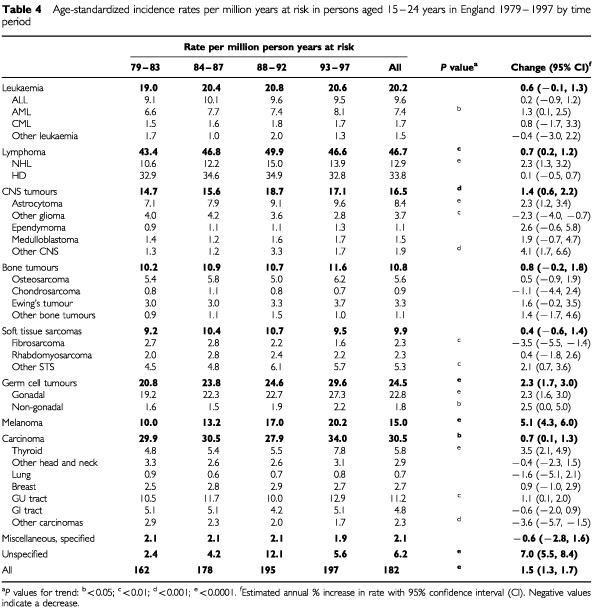
Age-standardized incidence rates per million years at risk in persons aged 15–24 years in England 1979–1997 by time period

presents time trends in incidence rates for the whole age group (15 to 24 years). Across all diagnostic groups there was a highly significant increase in incidence rates over time (*P*<0.0001), although there was little difference between the third and fourth time periods. Rates for leukaemias overall remained stable over the study period, but AML showed a small increase. Lymphomas overall showed a significant increase which was entirely accounted for by NHL.

Rates for CNS tumours increased over time. Among sub-groups of brain tumours, astrocytoma and ‘other CNS’ tumours showed increases but the ‘other glioma’ group showed a significant decrease. Rates for all bone tumours did not change during the study period. Rates for soft tissue sarcomas overall, and for rhabdomyosarcoma also did not change but rates for fibrosarcoma decreased, while the incidence of other soft tissue sarcomas increased.

Very marked increases in incidence were seen for gonadal germ cell tumours, melanoma and carcinoma of the thyroid. The increase in gonadal germ cell tumours was accounted for by testicular tumours (average annual percentage increase 9.5, 95% CI 3.1, 16.3; *P* for trend <0.0001). There was no corresponding increase in ovarian germ cell tumours (*P*=0.2).

There was a small but significant increase in rates for GU tract carcinomas. Further examination of this latter group showed that the increase was mainly due to ovarian carcinoma (average annual percentage increase 3.0, 95% CI 1.2, 4.9; *P*=0.001).

The rates for melanoma include melanoma of skin and non-skin sites mainly ocular (67 cases). The observed increase in incidence was entirely due to melanoma of skin and was consistent across both age groups and both sexes. The pattern of melanoma of skin in males and females was examined according to primary site (head and neck, trunk, upper limbs, lower limb). There was a highly statistically significant interaction between site and sex, (*P*<0.0001). Males had a slightly higher rate of head and neck melanoma but females had a higher rate of trunk melanoma and much higher rates of limb (particularly lower limb) melanoma. Rates for melanoma of skin of lower limb were 2.6 per million in males and 9.1 per million in females. The relative changes in site distribution of melanoma over time did not reach statistical significance (*P*=0.06).

Carcinomas of other and ill-defined sites showed a decrease in incidence over time which is probably due to improvements in the quality of cancer registration, but the numbers are relatively small and do not account for the observed increases in specified groups of carcinomas. Unspecified malignant neoplasms NEC (Group 10) showed a significant increase in incidence over time and rates during 1988–92 were particularly high. More than one third of all cases in this group were coded to G.U. sites. The most frequent site was testis. It is likely that the majority of these would be germ cell tumours. The reason for this large number of unspecified registrations during this period is unclear but registrations originally by death certificate, which were retrospectively allocated to a year of diagnosis, may be a contributory factor (Quinn *et al*, 2001).

## DISCUSSION

The need for a morphology-based classification system for presentation of data on cancers in children was recognised some time ago (Birch and Marsden, 1987; Kramárová and Stiller, 1996). This childhood cancer classification has been applied to cancer incidence data in adolescents aged 15 to 19 years (Smith *et al*, 1999). However, this is not appropriate, since a number of the major childhood cancer groups are irrelevant in adolescents and young adults. Conversely, carcinomas, as specified in the childhood cancer classification, are inappropriately sub-divided and do not describe the pattern observed in young adults adequately (Fritschi *et al*, 1995). While leukaemias and lymphomas can be defined by ICD it is not possible to define soft tissue sarcomas and germ cell tumours, which arise in many different anatomical sites and ICD cannot distinguish non-epithelial cancers from carcinomas or different types of these from each other. For example, carcinomas, soft tissue sarcomas and germ cell tumours arising in the liver would all be allocated the code for malignant neoplasm of liver. Furthermore, there are important differences in the incidence patterns of morphological sub-types of CNS and bone tumours. Given these difficulties, Bleyer (2002) has suggested that a separate nosologic system should be used for cancers that occur in older adolescents and young adults to take account of the unique features in this age group.

The morphology-based scheme presented above has been developed specifically for the 15 to 24 year age range and the diagnostic groups reflect the numerical importance of cancers observed. A number of the defined groups in the above scheme are comparable to the equivalent groups in the childhood cancer classifications and Birch and Marsden, 1987; Kramarova and Stiller, 1996) thereby facilitating comparisons of rates in children with those in adolescents and young adults. However, it would also be of interest to apply the scheme to cancers in children aged 10–14 years, since in this age group embryonal tumours are relatively rare.

We should like to propose the use of this classification scheme in future studies of cancers in adolescents and young adults to achieve a standard format for presentation of such data, to facilitate international comparisons, and to encourage an interest in research into these cancers.

In children aged less than 15 years, 33% of all malignancies are leukaemias, 25% are CNS tumours, 9% are lymphomas, 9% are bone and soft tissue sarcomas, and childhood embryonal tumours (neuroblastoma, retinoblastoma, Wilms' tumour and hepatoblastoma), comprise 16%. Less than 2% of childhood cancers are carcinomas (Mcnally *et al*, 2001a,b). It can be seen (Table 3) that the distribution of cancer types among the 15 to 19 year olds represents a transitional pattern between that seen in children and the pattern seen in the 20 to 24-year-olds which is somewhat more typical of older ages. In both age groups, lymphomas have the highest rates, but whereas leukaemia has the second highest rate in the younger group, it drops to fifth in the older group. Similarly, CNS tumours and bone tumours also drop in rank order in the older group but carcinomas, germ cell tumours and melanoma increase in numerical importance. Childhood embryonal tumours comprised only 0.3% of all cancers in the 15–24 year age range.

In 15 to 24-year-olds, carcinomas represent 17% of total registrations but the primary site distribution is very different from that seen at older ages. Across all ages, cancers of lung, colon and rectum together, represent 28% of all malignancies (Quinn *et al*, 2001), but only 2% of malignancies in 15 to 24-year-olds are carcinomas of these sites. In contrast, in 15 to 24-year-olds, melanoma and carcinoma of the thyroid represent 8 and 3% of all cancers respectively, but across all ages these cancers make up only 2 and 0.4% of the total. One of the most striking patterns was the more than three-fold increase in incidence rates with age for germ cell tumours in males, largely due to testicular teratoma and seminoma. The incidence of testicular cancer overall continues to rise reaching a peak of around 130 per million in men aged 30 to 34, after which there is a sharp decline in rates with age (Quinn *et al*, 2001).

For most groups the pattern of ratios of rates in males to rates in females is similar in both the 15–19 year age group and the 20–24 year age group and is also similar to that found in children aged 10–14 years (Mcnally *et al*, 2001a,b; Stiller *et al*, 1998). Overall, and in most groups, rates were higher in males than females. In certain diagnostic groups however, the sex ratio differed in the two age groups notably germ cell tumours in which the rates in males increased markedly in the older group. This contrasts with the pattern in children where there is a higher rate of germ cell tumours in children aged 10–14 years. The noticably higher rate of melanoma in females in both the 15–19-year-olds and the 20–24-year-olds is not seen in 10–14-year-olds, but the overall rates are very much lower in the latter age group (Mcnally *et al*, 2001a; Stiller *et al*, 1998). Other groups showing marked increases in rates with age in females include carcinomas of thyroid, breast and GU tract. These cancers are extremely rare in children but a female excess of carcinoma of the thyroid in children is nevertheless apparent (Stiller *et al*, 1998). Another of the more marked changes with age occurs in HD. In children aged 10–14 the male to female ratio for HD is 1.8 (Mcnally *et al*, 2001b). In adolescents and young adults this ratio becomes 1.0. This change in the sex ratio in HD may reflect differing aetiologies and proportions of sub-types at different ages.

When incidence rates in the two age groups combined were examined for trends over time, marked increases in rates were seen for all cancers together in the second and third periods. The largest increases were seen for melanoma of skin, where the incidence doubled from the earliest period to the latest period, and in germ-cell tumours, which increased by 50%. There were also highly significant increases in the incidence of NHL, CNS tumours and certain carcinomas.

Increases in the incidence of melanoma of skin and testicular cancer in young adults have been noted previously (Quinn *et al*, 2001) but although increases in the incidence of NHL have been described (Quinn *et al*, 2001; Mcnally *et al*, 1999) these were accounted for mainly by disease occurrence at older ages. There was no change in rates for HD and while there was a small increase in AML, there was no change in the incidence of ALL. These trends for leukaemias and lymphomas contrast with those recently reported in children where there were significant increases in ALL and HD but stable rates for AML and NHL (Mcnally *et al*, 2001b). In ALL the increase in children was due to B-cell precursor disease and in HD the rise was accounted for by the nodular sclerosis sub-type. It is not possible to examine trends by sub-type in the current data since information on immunophenotype is not available and a large number (32%) of HD cases were not coded to a specific sub-type. However, the overall contrasting patterns in incidence trends suggest differences in aetiology among leukaemias and lymphomas in children, adolescents and young adults.

When CNS tumours were analysed by sub-type, the overall increase was found to be due to astrocytic tumours and miscellaneous brain tumours. There was a decrease in the ‘other glioma’ group and some, but not all of the increase in astrocytomas may be due to more specific classification of these other gliomas. The trend in astrocytoma is consistent with that recently reported for astrocytomas in children and common aetiological factors may be operating (Mcnally *et al*, 2001a). Further examination of the increase in miscellaneous brain tumours showed that the increase was accounted for by unspecified tumours from 1988 onwards. This may be due to additional cases being diagnosed as a result of better radiological techniques.

We observed an increase in the rates for thyroid carcinoma, particularly in females (data not shown). An increased incidence for thyroid cancer in young adults, has been noted before (Cotterill *et al*, 2001). Concern was raised that this may be related to radioactive fall-out from the Chernobyl accident, but the observed pattern of thyroid cancer incidence is inconsistent with this interpretation (Stiller, 2001). It has also been suggested that thyroid cancer is negatively associated with cigarette smoking. The higher rates in women were said to be due to lower rates of cigarette smoking compared with men (Kreiger and Parkes, 2000). However, this putative association would not account for the trends observed in the present study.

It is interesting to note the significantly increasing incidence of ovarian carcinoma in young women (data not shown) given the highly significant increase in testicular germ cell tumours but no comparable increase in ovarian germ cell tumours. Most of the observed increase in ovarian carcinomas occurred during the 1993–1997 period. The data for 1995–1997 were coded according to ICD-O2 which includes certain tumours classified as malignant which were formerly assigned to the ‘uncertain behaviour’ category in ICD-O1. We therefore examined the ovarian carcinoma data for the relevant codes. There were 48 such cases and if these are removed from the data, the trend is no longer significant. A similar observation was made for data on patients aged 15–19 years with ovarian carcinoma who were included in the SEER programme (Smith *et al*, 1999). The apparent increase in the incidence of ovarian carcinoma in adolescent and young adult women in England may therefore be artefactual. However, the coding changes may also reflect changes in diagnostic practice among pathologists and it is not certain that the 48 tumours would have been coded as having ‘uncertain behaviour’ in earlier years.

Among soft tissue sarcomas, fibromatous tumours showed a decrease in incidence but there was a corresponding rise in the incidence of miscellaneous soft tissue sarcomas. The observed trend in both of these groups is almost certainly due to changes in histopathological classification. The terms fibrosarcoma and fibrous histiocytoma are applied much more stringently in current histopathological practice than in the past. With the development of immunohistochemical techniques it is now possible to classify soft tissue sarcomas more precisely (Weiss and Goldblum, 2001).

The rates shown in Table 3 are largely consistent with those found in a small study in this age group in the Northern Region of England and which partially overlaps with the present study (Cotterill *et al*, 2000). However, the Northern Region study included less than one tenth the number of cases in the present study and variations in rates with respect to diagnostic sub-groups are probably due to the very small numbers resulting in unstable rates for a number of the groups. For example, the rate for rhabdomyosarcoma in the Northern Region in 20–24-year-olds was reported as 0.8 per million compared with 1.4 per million in the present study, but the former rate was based on only five cases. In a more substantial series of cancers in 15–19-year-olds included in the SEER Programme, Smith *et al* (1999) found an overall cancer rate of 202 per million compared with 144 in the present study. Rates for leukaemia, HD and rhabdomyosarcoma were very similar but higher rates for most other cancers were seen in the SEER data. This was particularly marked for germ cell tumours, thyroid carcinoma and malignant melanoma. These are all cancers which have shown increases in incidence over time (Table 4 and Smith *et al*, 1999) which may well be related to changes in socio-economic and lifestyle factors. Such effects may have been operating in the United States earlier than in England resulting in the higher rates in the SEER series.

In conclusion, we have devised a classification scheme specifically tailored to cancers in adolescents and young adults which we hope will be used by others. The management of cancer in this age group should address patients' requirements for further and higher education, vocational and professional training, social and career development and possible long term treatment-related morbidity including preservation of fertility (Lewis, 1996). The data presented on cancer incidence will provide a basis for service planning, including support services for survivors, which will address the special needs of this patient group. We hope that the data, in particular the increasing incidence trends in specific diagnoses, will stimulate interest in devising targeted studies among young cancer patients. Future studies should focus on possible aetiological factors.

## References

[bib1] American Cancer Society1968 Manual of tumor nomenclature and codingNew York

[bib2] BirchJMMarsdenHB1987 A classification scheme for childhood cancer Int J Cancer 40 624629 10.1002/ijc.2910400508 3679589

[bib3] BlairVBirchJM1994 Patterns and temporal trends in the incidence of malignant disease in children: I. leukaemia and lymphoma Eur J Cancer 30 14901498 10.1016/0959-8049(94)00274-9 7833108

[bib4] BleyerWA2002 Cancer in older adolescents and young adults: epidemiology, diagnosis, treatment, survival and importance of clinical trials Med Pediatr Oncol 38 110 11835231 10.1002/mpo.1257

[bib5] CotterillSJParkerLMalcolmAJReidMMoreLCraftAW2000 Incidence and survival for cancer in children and young adults in the North of England, 1968–1995: a report from the Northern Region Young Persons' Malignant Disease Registry Br J Cancer 833397403 10917558 10.1054/bjoc.2000.1313 PMC2374562

[bib6] CotterillSJPearceMSParkerL2001 Thyroid cancer in children and young adults in the North of England. Is increasing incidence related to the Chernobyl accident? Eur J Cancer 37 10201026 11334728 10.1016/s0959-8049(00)00449-4

[bib7] Department of Health2000 The NHS Cancer Plan. A plan for investment. A plan for reformLondon: Department of Health

[bib8] FrancisBGreenMPayneC1993 The GLIM system, Release 4 ManualOxford: Clarendon Press

[bib9] FritschiLCoatesMMcCredieM1995 Incidence of cancer among New South Wales adolescents: Which classification scheme describes adolescent cancers better? Int J Cancer 60 355360 7829244 10.1002/ijc.2910600314

[bib10] KramárováEStillerCA1996 The International Classification of Childhood Cancer Int J Cancer 68 759765 8980180 10.1002/(SICI)1097-0215(19961211)68:6<759::AID-IJC12>3.0.CO;2-W

[bib11] KreigerNParkesR2000 Cigarette smoking and the risk of thyroid cancer Eur J Cancer 36 19691973 11000579 10.1016/s0959-8049(00)00198-2

[bib12] LewisIJ1996 Cancer in adolescence Br Med Bull 52 887897 9039738 10.1093/oxfordjournals.bmb.a011589

[bib13] McNallyRJQRomanECartwrightRA1999 Leukaemias and lymphomas: Time trends in the UK, 1984–93 Cancer Causes and Control 10 3542 10334640 10.1023/a:1008859730818

[bib14] McNallyRJQKelseyAMCairnsDPTaylorGMEdenOBBirchJM2001a Temporal increases in the incidence of childhood solid tumors seen in northwest England (1954–1998) are likely to be real Cancer 92 19671976 11745272 10.1002/1097-0142(20011001)92:7<1967::aid-cncr1716>3.0.co;2-#

[bib15] McNallyRJQCairnsDPEdenOBKelseyAMTaylorGMBirchJM2001b Examination of temporal trends in the incidence of childhood leukaemias and lymphomas provides aetiological clues Leukemia 15 16121618 11587220 10.1038/sj.leu.2402252

[bib16] ParhamDM(eds)1996 Ewings's sarcoma, peripheral neuroepithelioma and related tumorsChapter 4In:Pediatric neoplasia: morphology and biologyPhiladelphia: Lippincott-Raven

[bib17] ParkinDMWhelanSFerlayJRaymondLYoungJ(eds)1997 Cancer incidence in five continents,Vol VIILyon: IARC (IARC Scientific Publications no 143)

[bib18] PercyCVan HoltenVMuirC(eds)1990 International classification of diseases for oncology (ICD-O)2nd edn.Geneva: World Health Organization

[bib19] QuinnMJBabbPJJonesJBakerAAultC1999Cancer 1971–1997: Registrations of cancer cases and deaths in England and Wales by sex, age, year, health region and type of cancer (CD-ROM).London: Office for National Statistics

[bib20] QuinnMBabbPBrockAKirbyLJonesJ2001 Cancer trends in England and Wales. 1950–1999Studies on medical and population subjects No 66.London: The Stationery Office

[bib21] SmithMAGurneyJGRiesLA1999 Cancer in adolescents 15–19 years oldInCancer incidence and survival among children and adolescents.Ries LA, Smith MA, Gurney JG, Linet M, Tamra T, Young JL, Bunin GR (eds)United States SEER Program 1975–1997, National Cancer Institute, SEER ProgramBethesda, MD: NIH Pub No 99-4649

[bib22] StillerCA2001 Thyroid cancer following Chernobyl Eur J Cancer 37 945947 11334718 10.1016/s0959-8049(01)00072-7

[bib23] StillerCAAllenMBayneABrownbillPDraperGEatockELoachMVincentT(eds)1998 United Kingdom National Registry of Childhood Tumours, England and Wales, 1981–1990InInternational Incidence of Childhood CancerVol II.Lyon: IARC (IARC Scientific Publications no 144)

[bib24] WalshPMComberHGavinAT2001 All-Ireland Cancer Statistics 1994–96. A joint report on incidence and mortality for the island of IrelandNational Cancer Registry (Ireland), Cork and Northern Ireland Cancer Registry: Belfast

[bib25] WeissSWGoldblumJR2001 Enzinger and Weiss's soft tissue tumours4th edition.Mosby: St Louis

[bib26] World Health Organisation1975 International statistical classification of diseases, injuries and causes of death9th Revn.Geneva: WHO

[bib27] World Health Organisation1976 International classification of diseases for oncology1st edn.Geneva: WHO

[bib28] World Health Organisation1992 International statistical classification of diseases and related health problems10th Revn. Geneva: WHO

